# Stability Analysis of a High Fibre Yield and Low Lignin Content “Thick Stem” Mutant in Tossa Jute (*Corchorus olitorius* L.)

**DOI:** 10.1155/2014/539869

**Published:** 2014-04-22

**Authors:** Aninda Mandal, Animesh K. Datta

**Affiliations:** Department of Botany, Cytogenetics, Genetics and Plant Breeding Section, Kalyani University, Kalyani, West Bengal 741235, India

## Abstract

A “thick stem” mutant of* Corchorus olitorius* L. was induced at M_2_ (0.50%, 4 h, EMS) and the true breeding mutant is assessed across generations (M_5_ to M_7_) considering morphometric traits as well as SEM analysis of pollen grains and raw jute fibres, stem anatomy, cytogenetical attributes, and lignin content in relation to control. Furthermore, single fibre diameter and tensile strength are also analysed. The objective is to assess the stability of mutant for its effective exploration for raising a new plant type in tossa jute for commercial exploitation and efficient breeding. The mutant trait is monogenic recessive to normal. Results indicate that “thick stem” mutant is stable across generations (2*n* = 14) with distinctive high seed and fibre yield and significantly low lignin content. Stem anatomy of the mutant shows significant enhancement in fibre zone, number of fibre pyramids and fibre bundles per pyramid, and diameter of fibre cell in relation to control. Moreover, tensile strength of mutant fibre is significantly higher than control fibre and the trait is inversely related to fibre diameter. However the mutant is associated with low germination frequency, poor seed viability, and high pollen sterility, which may be eliminated through mutational approach followed by rigorous selection and efficient breeding.

## 1. Introduction

Induction of mutation forms an integral part of breeding program as it widens the gene pool through creation of genetic variability. The methodology has been successfully adapted for crop improvement and release of elite “plant type” mutants [1] including* Corchorus olitorius* (tossa jute; family: Tiliaceae), an important fibre yielding crop of commerce [2–5]. A “thick stem” (designated as “thick stem I”) mutant was isolated from M_2_ mutagenised population of* C. olitorius* var. JRO 524 following seed treatment with ethyl methane sulphonate (EMS) and the mutant bred true at M_4_ generation [6]. Present investigation describes the “thick stem” mutant in relation to control considering morphometric traits as well as scanning electron microscopic (SEM) analysis of pollen grains and raw jute fibres, anatomical (stem anatomy) attributes, cytogenetical (meiosis and inheritance of the mutant trait) parameters, and biochemical (lignin and holocellulose contents) aspects in advanced generations (M_5_ to M_7_) under uniform environmental conditions. Furthermore, single fibre diameter and tensile strength are also analysed. The objective of the work is to assess the stability of the mutant across generations for its effective exploration in raising a new “plant type” as well as in efficient breeding in tossa jute.

## 2. Materials and Methods

### 2.1. Germplasms

Selfed seeds of true breeding M_4_ “thick stem” mutant (raised at M_2_ following 0.50%, 4 h EMS treatment) as well as selfed control lines (seeds of 5 phenotypically stable plants in each case were considered in each generation) of* C. olitorius* L. var. JRO 524 (mother seed stock obtained from Central Research Institute for Jute and Allied Fibres, West Bengal, India, in the year 2006) were sown in randomised block design with 3 replications (plot size: 3 m×1.5 m; between plants 30 cm and rows 20 cm). Plants were grown in experimental field plots of Kalyani University (West Bengal plain, Nadia: latitude 22°50′ to 24°11′ N, longitude 88°09′ to 88°48′ E, altitude 9.75 m; sandy loamy soil, soil pH 6.85) from March to October 2010, 2011, and 2012 (M_5_ to M_7_). No fertilizer application was made during the growth period of the plant types.

### 2.2. Study of Quantitative Parameters

Germination frequency (Petri plates as well as field; data for field is pooled over the plots) and morphometric traits assessed are presented in Table 1. On an average 15 to 20 randomly selected plants were studied for quantitative parameters in each plant type, in each generation. Quantitative parameters were studied on harvest. Fibre yield in the plant types was estimated following the conventional method of whole plant retting. For the purpose, defoliated plants of mutant and control were dipped in stagnant water in a cistern (artificially prepared). Brick bats tied in cement bags were used to deep the jak materials. Retting was completed within 15 to 20 days. Fibres were extracted by “beat-break-jerk” method. The fibres extracted from a single plant were sun-dried before weighing.

Seed viability was tested using 1% tetrazolium chloride following the method suggested by Patil and Dadlani [7].

### 2.3. Girth of Stem and Anatomical Attributes

Girth of stem was assessed in both the plant types uniformly from base, middle, and upper zones. Stem anatomical features (Table 3) of “thick stem” mutant (assessed at M_7_) in relation to control were analysed from suitable transverse sections from base, middle, and upper portions. The sections were stained following the method described by Johansen [8]. Photomicrographs were taken from suitable sections.

### 2.4. Meiotic Analyses

For the meiotic studies, 3 to 5 floral buds of suitable size from 3 randomly selected plants of both plant types were fixed (6 am to 7 am) in each generation (M_5_ to M_7_) in Carnoy's fixative (6 ethanol : 3 chloroform : 1 acetic acid). Two changes at an interval of 24 h were given in the fixative and preserved in 70% alcohol under refrigerated condition. Pollen mother cells (PMCs) and pollen grains obtained from anther squash preparations were stained in 2% propinocarmine solution. Fully stained pollen grains were considered fertile [9]. Data were scored from scattered diplotene-diakinesis, metaphase I (MI), and anaphase I (AI) meiocytes and pooled over the buds in each plant type. Photomicrographs were taken from temporary slide preparation and subsequently magnified.

### 2.5. Study of Inheritance Pattern

At anthesis (9 am to 11 am) reciprocal crossings between control and “thick stem” mutant (20 crosses in each set) were performed considering all necessary precautionary measures to avoid genetic contaminations as well as autogamy. Hybrid seeds obtained were sown to raise F_1_ plant population. The F_1_ plants were selfed (5 floral buds were bagged at the onset of anthesis in each category) and seeds obtained were sown to raise F_2_ plants. The F_2_ plants segregated into normal and mutant phenotypes and *χ* ^2^ test analysis was performed to assess segregation pattern.

### 2.6. SEM Analyses

Surface morphology of pollen grains (matured pollen grains from fully opened flowers) and raw jute fibres of control and mutant was studied under SEM (Zeiss EVO HD, Oberkochen, Germany) at 15 kV accelerating voltage at GSI (Geological Survey of India, Kolkata, West Bengal, India). Photomicrographs were taken from suitable preparations. Pollen shape and size were determined as per Erdtman [10].

### 2.7. Biochemical Analyses

Lignin content was analysed in control and in “thick stem” mutant (5 plants of each plant type were considered) from retted fibres of harvested M_7_ plants as per Sengupta et al. [11]. For the purpose, dry milled fibres were thoroughly cleaned of wax and oil in a Soxhlet apparatus using a mixture of benzene and ethanol (2 : 1 v/v) and dried under vacuum. The samples (5 g in each case) were treated with 0.7% sodium chloride solution at pH 4.0, maintained by acetic acid for 2 h in a boiling water bath. The process was repeated 3 times and the samples were brought to neutral pH by washing with 2% sodium metabisulphite and water at 60°C, cooled and dried in desiccators to a constant weight to get the amount of holocellulose. The lignin content was determined by subtracting the amount of holocellulose from that of the dewaxed samples.

### 2.8. Diameter of Fibre

Fibres extracted from untreated control and mutant (M_7_ harvested plants) plant types were polygonal in shape and nonuniform in thickness. The fibres were considered as perfectly cylindrical to simplify measurements in accordance to de Rosa et al. [12]. Each fibre was manually separated from both plant types and measured in a light microscope at 10x magnification and diameter was calculated. Randomly 25 fibres from each set (control and mutant) were analysed. Uniformly 60 mm stretch of each fibre was tested. For each fibre, an average of 3 measurements (mean represented) of diameter at 3 different regions were taken as the diameter of that fibre.

### 2.9. Tensile Strength

Fibre strength was determined from the machine “Fibre bundle strength tester” following the methodology adopted by Bandyopadhyay and Mukhopadhyay [13]. The formula used to calculate Fibre bundle strength, which is (Breaking load in kilogram × length of bundle in cm × 10)/Sample weight in milligram, is expressed in g/tex. For the purpose, 10 bundles from each plant type were assessed.

### 2.10. Statistical Analyses

Student * t*-test was performed between control and mutant to assess significant variations, if any, considering morphometric traits (pooled over the generations) and anatomical attributes. Furthermore, *χ* ^2^ test of heterogeneity was conducted for each quantitative parameter to ascertain stability across the generations.

## 3. Results

Seed germination frequency across the generation was noted to be 84.0% to 86.0% and 68.0% to 72.0% in control and 28.0% to 32.0% and 18.0% to 20.0% in “thick stem” mutant under Petri plate and field conditions, respectively. Percentage of seed viability was also found to be much lower in mutant (10.0% to 15.0%) than control (96.0% to 98.0%).

### 3.1. Analyses of Quantitative Parameters

**Figure 1:**
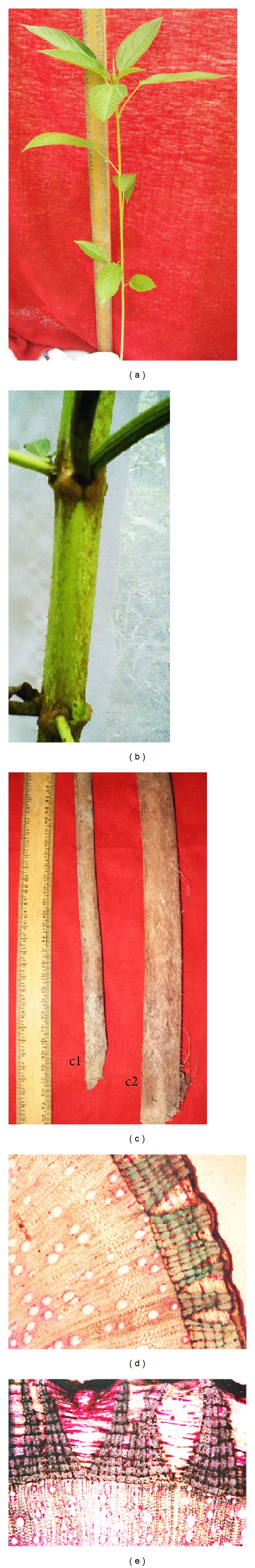
Tossa jute plant types ((a), (b)), sticks (c), and stem anatomy of middle zone ((d), (e)). ((a), (d))* Corchorus olitorius*. ((b), (e)) “thick stem” mutant. (c) Jute sticks after retting (control: c1, mutant: c2).

Data analysed for morphometric traits of control (Figure 1(a)) and “thick stem” mutant (Figure 1(b)) across the generation are presented in Table 1. Results indicated that “thick stem” mutant in relation to control shows significant (*P* < 0.01 to 0.001) enhancement of plant height, total number of branches per plant, total capsules per plant, seeds per capsule, seed yield per plant, and fibre yield per plant. Fibre yield per plant was convincingly higher in the mutant (78.33 g ± 1.67 to 80.12 g ± 1.78) than control (24.28 g ± 1.10 to 25.00 g ± 0.50). The quantitative traits were found to be randomly distributed (*P* > 0.05, DF 2) across the generations in both plant types.

### 3.2. Girth of the Stem and Anatomical Features

Anatomical attributes of control and mutant plant types are presented in Tables 2 and 3. Girth of stem of mutant was significantly thicker in base, middle, and upper zone than control (Table 2, Figure 1(c)). Study of transverse sections of stem of the mutant from base, middle, and upper portions revealed significant (*P* < 0.001) enhancement in fibre zone, number of fibre pyramids per section, number of fibre bundles per pyramid, and diameter of fibre cell in relation to control plant type (Table 3, Figures 1(d)–1(e)).

### 3.3. Meiotic Analyses

**Figure 2:**
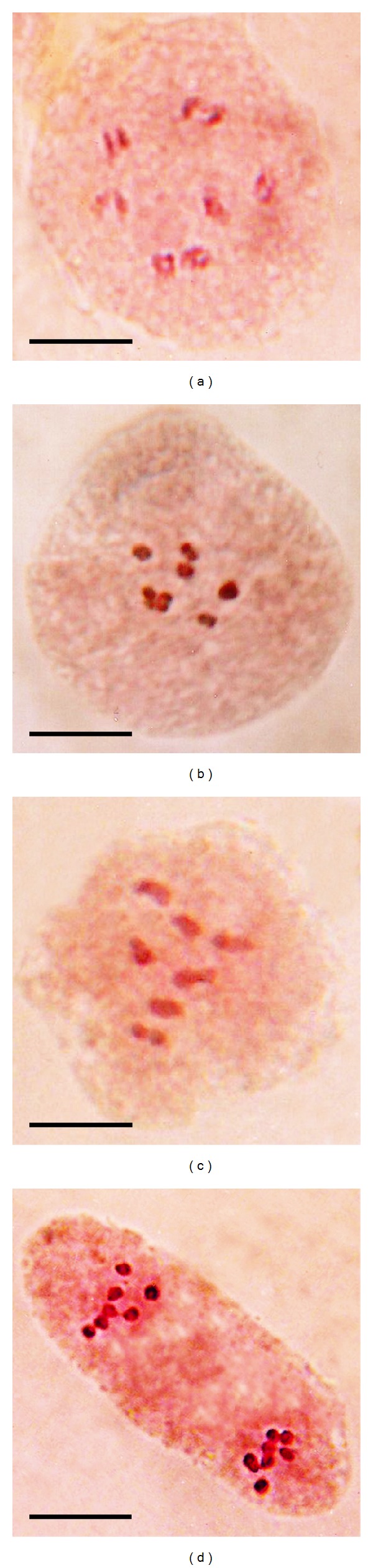
Meiotic configurations in control and mutant plant types at diplotene (a), metaphase I ((b), (c)) and anaphase I (d). ((a)–(c)) 7II. (d) 7/7 separation of chromosomes. Scale bar = 10 *μ*m.

Meiotic configurations and pollen fertility of the plant types are presented in Table 1. The meiocytes of both control and mutant showed 2*n* = 14 chromosomes always (Figures 2(a)–2(d)). Assessment of PMCs at diplotene indicated that bivalents were predominantly of rod configuration. Chiasma frequency per cell was noted to be 7.27  ±  0.10 (7.09  ±  0.12 to 7.42  ±  0.09) in control and 7.05 ±  0.08 (6.97 ±  0.07 to 7.17 ±  0.07) in the mutant. Bivalents formed at MI were randomly (*P* > 0.05, DF 2) distributed across generations in the plant types. Formation of 7II in PMCs at MI was always 100% in mutant and it varied from 80.77% to 88.46% (pooled 85.45%) in control. Equal (7/7) segregation of chromosomes at AI was found to be 100% in the plant types (Figure 2(d)). However, pollen fertility was recorded to be higher in control than the mutant.

### 3.4. Crossings and F_2_ Segregation

Out of 20 reciprocal crosses performed, only a few (4 in case—mutant as stigma parent and control as pollen parent; only 1 in opposite set) were successful in terms of fruit formation and seed setting. The hybrid capsules were smaller in size (3.84 cm to 5.12 cm) than either of the parents (control: 8.10 cm ± 0.6 to 8.20 cm ± 1.2; mutant: 7.82 cm ± 0.28 to 8.00 cm ± 0.50) and yielded much lesser number of seeds (33 to 47 per capsule) than parents (control: 180 ± 3.8 to 185 ± 2.0 per capsule; mutant: 204 ± 3.8 to 210 ± 3.54 per capsule).

F_1_ plants in all sets of crossings were phenotypically similar to normal plants. F_2_ plants were found to segregate into 3 : 1 (control as pollen parent × mutant as stigma parent: total 41, normal 32, mutant 09, *χ* ^2^ = 0.20 at 1 DF, *P* > 0.50; reciprocal: total 29, normal 23, mutant 06, *χ* ^2^ = 0.22 at 1 DF, *P* > 0.60) ratio.

### 3.5. SEM Analysis

#### 3.5.1. Pollen Grains

**Figure 3:**
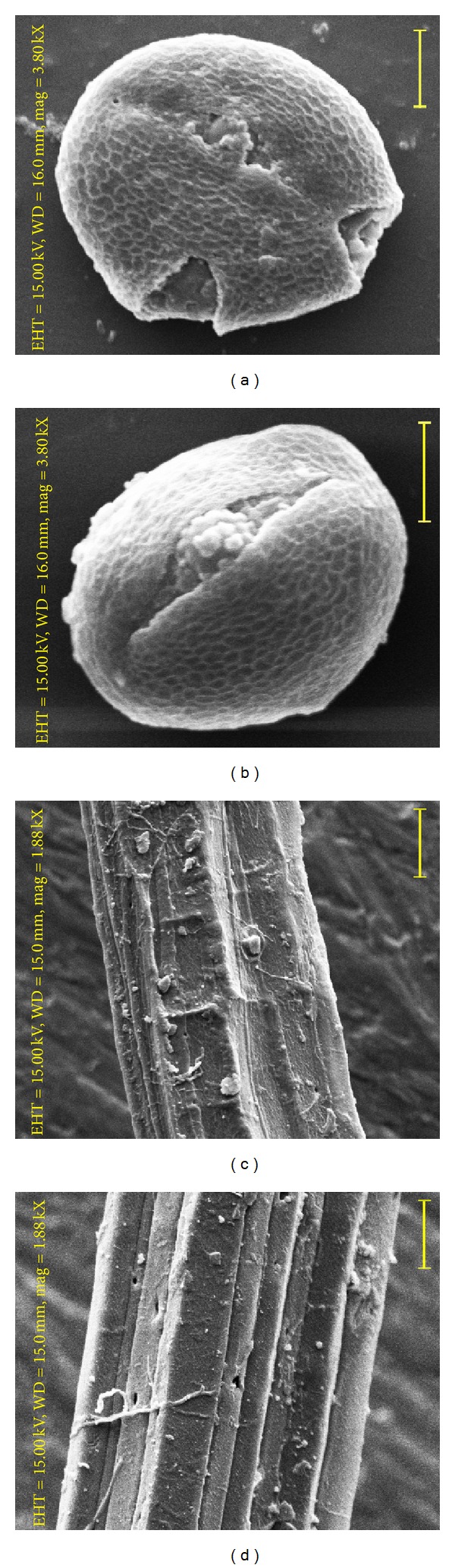
Scanning electron micrographs of pollen grains ((a), (b)) and raw jute fibres ((c), (d)). ((a) and (c))* Corchorus olitorius*. ((b) and (d)) “thick stem” mutant. Scale bar = 20 *μ*m.

In both the plant types pollen grains were subprolate; tricolporate; medium sized (control: 42.0 *μ*m ±1.6 × 36.0 *μ*m ± 0.8; mutant: 32.0 *μ*m ± 1.1 × 26.0 *μ*m ±0.7); colpi long (control: 33.0 *μ*m ± 1.1; mutant: 24.0 *μ*m ± 0.24), extending up to poles, wide, and asymmetrical; pore diameter 5.62 *μ*m  ±  0.3 (control) to 5.10 *μ*m  ±  0.2 (mutant); exine thick, reticulate, reticulation not uniform in size, becoming smaller towards the colpi margin; lumen area ranges from 0.22–1.60 *μ*m^2^ (control) to 0.18–1.32 *μ*m^2^ (mutant), polygonal; muri 0.38 *μ*m ± 0.15 (control) to 0.30 *μ*m ± 0.01 (mutant) thick (Figures 3(a) and 3(b)).

#### 3.5.2. Fibre

The raw jute fibre surface of both plant types following SEM are shown in (Figures 3(c) and 3(d)). The surface of jute fibre in control was rough, multicellular, and unevenly angular with verrucose lines and covered by impurities. Fibres of “thick stem” mutant were with longitudinal ridges and furrows. Ridges were not of equal breadth, covered by impurities. Fibres of both the plant types were pitted.

### 3.6. Biochemical Studies

The amount of holocellulose was found to be relatively higher in mutant (94.0% to 96.0%) than control (88.0% to 89.0%) plant type. Lignin content in fibre was estimated to be significantly (*P* < 0.001, DF 8) lower in the mutant (4.0% to 4.5%) than control (10.5% to 12.0%).

### 3.7. Physical Properties of Fibre

Fibre diameter was significantly (*t* = 8.614, DF = 48, *P* < 0.001) higher in control (0.1234 mm ± 0.007; range: 0.046 mm to 0.174 mm) than mutant (0.0514 mm ± 0.005; range: 0.026 mm to 0.096 mm). Tensile strength was 27.112  g/tex ± 0.196 in control (range: 26.40 g/tex to 28.10 g/tex) in comparison to 33.235 g/tex ± 0.227 in mutant (range: 32.20 g/tex to 34.20 g/tex) plant types. Tensile strength varied between the plant types significantly (*t* = 20.41, DF = 18, *P* < 0.001).

## 4. Discussion

Results indicate that “thick stem” mutant is stable across the generations in relation to assessed parameters. The mutant resembles control in meiotic chromosome behavior but pollen fertility is rather less. The mutant trait is found monogenic recessive to normal. The “thick stem” plant type yields higher amount of seeds and fibre per plant in comparison to control. Basu [2] reported 4 high fibre yielding “thick stem” mutants in* Corchorus* (2 each in* C. capsularis* and* C. olitorius*) following seed treatments with X-rays and *β*-rays and repeated selection. The mutants were early flowering with short flowering period. “Thick stem” mutants have also been reported in other plant species [14–16]. Enhancement in biomass density in stem tissue in dicotyledonous plants resulting in thick stem is reported in* Medicago truncatula* and* Arabidopsis thaliana* to be due to disruption of stem-expressed WRKY transcription factor (TF) genes, which consequently upregulate downstream genes encoding the NAM, ATAF1/2, and CUC2 (NAC) and CCCH type (C3H) zinc finger TFs that activate secondary wall synthesis [17].

Anatomical features reveal secondary xylem activity in “thick stem” mutant along with significant enhancement in fibre zone, number of fibre pyramids and fibre bundles per pyramid, and diameter of fibre cell in relation to control. SEM analysis reveals distinct variations in raw jute fibres of control and mutant plant types; however, both are pitted and with impurities. Optical measurement of diameter of a single fibre and assessment of tensile strength reveal that mutant fibres are much thinner with characteristically higher tensile strength than control fibres. Both the traits are significant for economic worth in jute [18, 19]. Average tensile strength noted in “thick stem” mutant is much higher than the reported [20] tensile strength in* C. olitorius* germplasms (JRO 632–25.5 g/tex, JRO 525–26.0 g/tex, JRO 7835–26.9 g/tex, JRO 66–26.6 g/tex, JRO 872–26.1 g/tex, JRO 66–26.6 g/tex, and JRO 128–27.5 g/tex, among others). Rashed et al. [21] reported that tensile strength increases proportionally with fibre size and fibre percentage; however, after a certain size and percentage the tensile strength decreases again. Alkaline treatments are reported to enhance tensile strength in jute [22, 23]. Inverse relationship noted between fibre diameter and tensile strength in the described mutant signifies its economic value in commerce.

The mutant also possesses low amount of lignin in retted fibres, a character of potential economic benefits. Most of the mutants and transgenic plants reported with modified lignin are xylem mutants [24–26]. Predominance of xylem mutants is possibly due to that wood is important for industrial processing, and also because tracheary elements are easier to detect than the phloem elements [27]. Furthermore, phenotypic screening for phloem mutants is reported to be rather difficult as lignin-deficient sclerenchyma cells of phloem tissue do not collapse [28] as easily as in tracheary cells [29]. Sengupta and Palit [30] reported a deficient lignified phloem fibre (dlpf) mutant in* C. capsularis* and the developmental deficiency has been attributed to phenylalanine lyase activity rather than peroxidase activity. Meshram and Palit [31] suggested that application of GA biosynthetic inhibitor helped to reduce lignin synthesis and to increase fibre fineness, prerequisite for fibre quality.

The mutant induced and maintained through generations possesses traits like high tensile strength and low lignin content in fibre and high fibre and seed yield per plant, which are extremely important for economic value of jute as fibre crop. However, the mutant is also associated with poor germination frequency, low seed viability, and high pollen sterility. Considering the plantation methodology adopted, germination frequency, and fibre yield per plant, it is expected that fibre yield will be about 3.18 ton/hectare in control and 2.76 ton/hectare in mutant but such variation is rather compensating due to significantly higher seed yield in mutant than control.

## 5. Conclusion

The “thick stem” mutant is an important genetic resource in* C. olitorius* and before its commercial exploitation the undesirable traits may be eliminated through mutational approach followed by rigorous selection or through efficient breeding.

## Figures and Tables

**Table 1:** Morphometric traits and meiosis in control and in mutants.

Attributes	Plant types							
	Control				“Thick stem”			
	2010	2011	2012	Pooled	*M*_ 5_	*M*_ 6_	*M*_ 7_	Pooled
Morphometric								
Plant height (cm)	262.25 ± 7.8	263.40 ± 7.0	264.00 ± 3.5	263.22 ± 6.10	315.50 ± 1.00	317.25 ± 1.00	316.40 ± 0.50	316.38 ± 0.83
Number of primary branches/plant	2.42 ± 0.4	2.40 ± 0.5	2.40 ± 0.4	2.41 ± 0.43	2.40 ± 0.40	2.00 ± 0.20	2.50 ± 0.20	2.27 ± 0.27
Total branches/plant	4.00 ± 0.5	4.10 ± 0.6	4.00 ± 0.5	4.03 ± 0.05	6.00 ± 0.30	5.50 ± 0.45	5.60 ± 0.40	5.70 ± 0.38
Number of capsule/plant	18.40 ± 0.3	18.48 ± 0.5	19.00 ± 0.1	18.63 ± 0.30	25.00 ± 0.80	22.67 ± 0.90	23.10 ± 1.00	23.59 ± 0.9
Capsule length (cm)	8.20 ± 1.2	8.20 ± 1.2	8.10 ± 0.6	8.17 ± 1.00	7.90 ± 1.10	7.82 ± 0.28	8.00 ± 0.50	7.91 ± 0.93
Seeds/capsule	180.00 ± 3.8	182.12 ± 2.6	185.00 ± 2.0	182.37 ± 2.80	210 ± 3.54	204 ± 3.80	207 ± 1.90	207 ± 3.08
Seed yield/plant (g)	6.80 ± 0.8	6.90 ± 0.4	7.18 ± 0.1	6.96 ± 0.43	7.98 ± 0.62	7.78 ± 0.40	7.85 ± 0.38	7.87 ± 0.47
Fiber yield/plant (g)	24.28 ± 1.1	25.00 ± 0.5	24.84 ± 1.0	24.71 ± 0.87	78.33 ± 1.67	80.00 ± 1.20	80.12 ± 1.78	79.48 ± 1.55
Meiosis								
Frequency/cell at diplotene								
I	0.12	0.48	0.39	0.33	0.71	0.76	0.57	0.68
II	6.94	6.75	6.80	6.86	6.64	6.62	6.72	6.66
Bivalent configurations								
Ring/cell	0.48 ± 0.12	0.33 ± 0.10	0.46 ± 0.12	0.43 ± 0.11	0.33 ± 0.11	0.38 ± 0.12	0.45 ± 0.10	0.39 ± 0.11
Rod/cell	6.46 ± 0.08	6.43 ± 0.06	6.34 ± 0.06	6.41 ± 0.07	6.31 ± 0.06	6.24 ± 0.05	6.27 ± 0.10	6.12 ± 0.06
Chiasma/cell	7.42 ± 0.09	7.09 ± 0.12	7.26 ± 0.10	7.27 ± 0.10	6.97 ± 0.12	6.97 ± 0.13	7.17 ± 0.08	7.05 ± 0.07
Number of cells scored at diplotene	104	79	102	285	42	63	60	165
Mean association/cell at MI								
I	0.42	0.36	0.31	0.37	0.00	0.00	0.00	0.00
II	6.97	6.82	6.85	6.82	7.00	7.00	7.00	7.00
Predominant association (7II) at MI (%)	86.05	80.77	88.46	85.45	100.00	100.00	100.00	100.00
Cells scored at MI	86	78	104	268	34	58	62	154
Equal (7/7) separation of chromosomes at AI (%)	100.00	100.00	100.00	100.00	100.00	100.00	100.00	100.00
Number of AI cells scored	33	17	22	72	11	17	13	41
Pollen fertility (%)	84.65	78.15	81.18	80.92	64.79	68.98	68.00	67.15
Number of pollen grains scored	482	737	919	2138	497	432	450	1379

**Table 2:** Girth of the stem in the plant types.

Attributes	Plant types								*t* value at 8 DF	*P* value
	Control				“Thick stem”			
	2010	2011	2012	Pooled	*M*_ 5_	*M*_ 6_	*M*_ 7_	Pooled
Girth of stem (cm)										
Basal	7.62 ± 0.47	7.87 ± 0.45	7.81 ± 0.20	7.77 ± 0.37	14.10 ± 0.37	13.90 ± 0.51	13.55 ± 0.48	13.85 ± 0.20	23.27	<0.001
Middle	5.77 ± 0.85	5.85 ± 0.70	5.72 ± 0.24	5.78 ± 0.60	12.20 ± 0.21	11.92 ± 0.45	12.10 ± 0.37	12.07 ± 0.34	24.49	<0.001
Upper	4.00 ± 0.43	3.47 ± 0.14	4.00 ± 0.37	3.82 ± 0.31	7.80 ± 0.37	7.60 ± 0.30	8.00 ± 0.37	7.80 ± 0.35	9.53	<0.001

**Table 3:** Stem anatomical attributes in the plant types of *C. olitorius*.

Attributes	Zones	Genotypes		*t* value at 8 DF	*P* value
		Control	“Thick stem”		
Fibre zone (cm^2^)	Base	0.53 ± 0.01	4.02 ± 0.09	16.43	<0.001
	Middle	0.30 ± 0.01	3.12 ± 0.07	11.87	<0.001
	Upper	0.21 ± 0.01	1.20 ± 0.05	21.50	<0.001
Number of fibre pyramid per T.S.	Base	60.20 ± 1.16	96.40 ± 0.51	28.60	<0.001
	Middle	50.80 ± 0.86	87.00 ± 0.71	32.50	<0.001
	Upper	45.20 ± 1.16	70.00 ± 0.71	18.30	<0.001
Number of fibre bundle per pyramid	Base	41.80 ± 1.43	75.40 ± 1.44	16.60	<0.001
	Middle	26.80 ± 0.80	57.80 ± 0.49	33.00	<0.001
	Upper	16.00 ± 0.32	31.00 ± 0.32	33.50	<0.001
Diameter of fibre cell (*µ*m)	Base	15.60 ± 0.25	18.60 ± 0.37	7.16	<0.001
	Middle	16.00 ± 0.32	18.80 ± 0.37	5.72	<0.001
	Upper	18.00 ± 0.32	21.20 ± 0.37	6.53	<0.001
